# Thoracoscopic Resection of a Giant Solitary Fibrous Tumor in the Chest Cavity After Preoperative Arterial Embolization: A Case Report

**DOI:** 10.7759/cureus.90612

**Published:** 2025-08-20

**Authors:** Ikki Takada, Yujin Kudo, Ryosuke Amemiya, Yuki Takara, Jun Matubayashi, Masayuki Uchida, Maki Ito, Tatsuo Ohira, Toshitaka Nagao, Kazuhiro Saito, Norihiko Ikeda

**Affiliations:** 1 Surgery, Tokyo Medical University, Tokyo, JPN; 2 Radiology, Tokyo Medical University, Tokyo, JPN; 3 Anatomic Pathology, Tokyo Medical University, Tokyo, JPN; 4 Anatomic Pathology, Tokyo Medical University Hospital, Tokyo, JPN

**Keywords:** arterial embolization, four-dimensional computed tomography, pleural tumor, solitary fibrous tumor, vats

## Abstract

Giant hypervascular solitary fibrous tumors of the pleura (SFTPs) present significant challenges due to their size and vascularity and potential involvement of adjacent structures. Careful preoperative planning of the surgical approach is required, depending on the tumor size and location. Furthermore, intraoperative bleeding may significantly complicate surgical procedures in patients with hypervascular tumors. Herein, we report a case of a giant hypervascular SFTP with a pulmonary artery shunt that was successfully managed by thoracoscopic excision after preoperative embolization of the vascular supplies.

A 60-year-old woman underwent computed tomography during a medical check-up. A mass measuring 12.6 cm in diameter was detected in the right thoracic cavity, which was extensively attached to the diaphragm. Magnetic resonance imaging revealed a slightly high signal on T1-weighted images and an extremely high signal on short-TI inversion recovery images. Four-dimensional computed tomography revealed that the right inferior phrenic artery was an arterial supply, and a branch of the middle lobar artery was contrasted as a draining vessel, forming a shunt. Angiography-guided embolization was performed to reduce vascularity, followed by an echo-guided percutaneous biopsy to confirm SFTP. The patient underwent three-port video-assisted thoracoscopic surgery. Intraoperatively, the cranial side of the tumor was found to be contiguous with the right middle lobe, requiring resection of the lobe together with the tumor. The caudal side exhibited abundant vascularity and was carefully dissected due to its proximity tothe diaphragm. Intraoperative frozen section diagnosis of the resected tumor revealed histological features suggestive of SFTP, consistent with the preoperative diagnosis, and no tumor cells were observed at either the right middle lobe or diaphragmatic margins, indicating a cranio-caudal R0 resection. The patient’s postoperative course was uneventful, and no recurrence was observed after two years.

Preoperative imaging demonstrated a hypervascular tumor in broad contact with the diaphragm, and the vascular pedicle was not clearly visualized due to compression by the tumor. Multiple feeding arteries were suspected; therefore, preoperative arterial embolization was performed to minimize intraoperative bleeding. A multidisciplinary strategy enabled safe and minimally invasive resection.

## Introduction

Solitary fibrous tumors of the pleura (SFTPs) are rare mesenchymal tumors that arise at various sites. They typically originate from the pleura, accounting for <5% of pleural tumors [1,2]. Although most SFTPs are benign, approximately 10-20% of cases demonstrate malignant features, which can result in local recurrence or metastasis [3]. Surgical resection is the standard treatment for SFTPs [4-6]. Nevertheless, large hypervascular tumors present distinct challenges, including the potential for substantial intraoperative bleeding. Preoperative assessment of tumor vascularity and embolization is crucial for optimizing surgical outcomes and minimizing complications, particularly in cases involving large and hypervascular tumors [7-9]. Herein, we report a case of a giant hypervascular SFTP, including a pulmonary artery shunt, which was successfully treated with preoperative arterial embolization and thoracoscopic surgery.

## Case presentation

**Figure 1:**
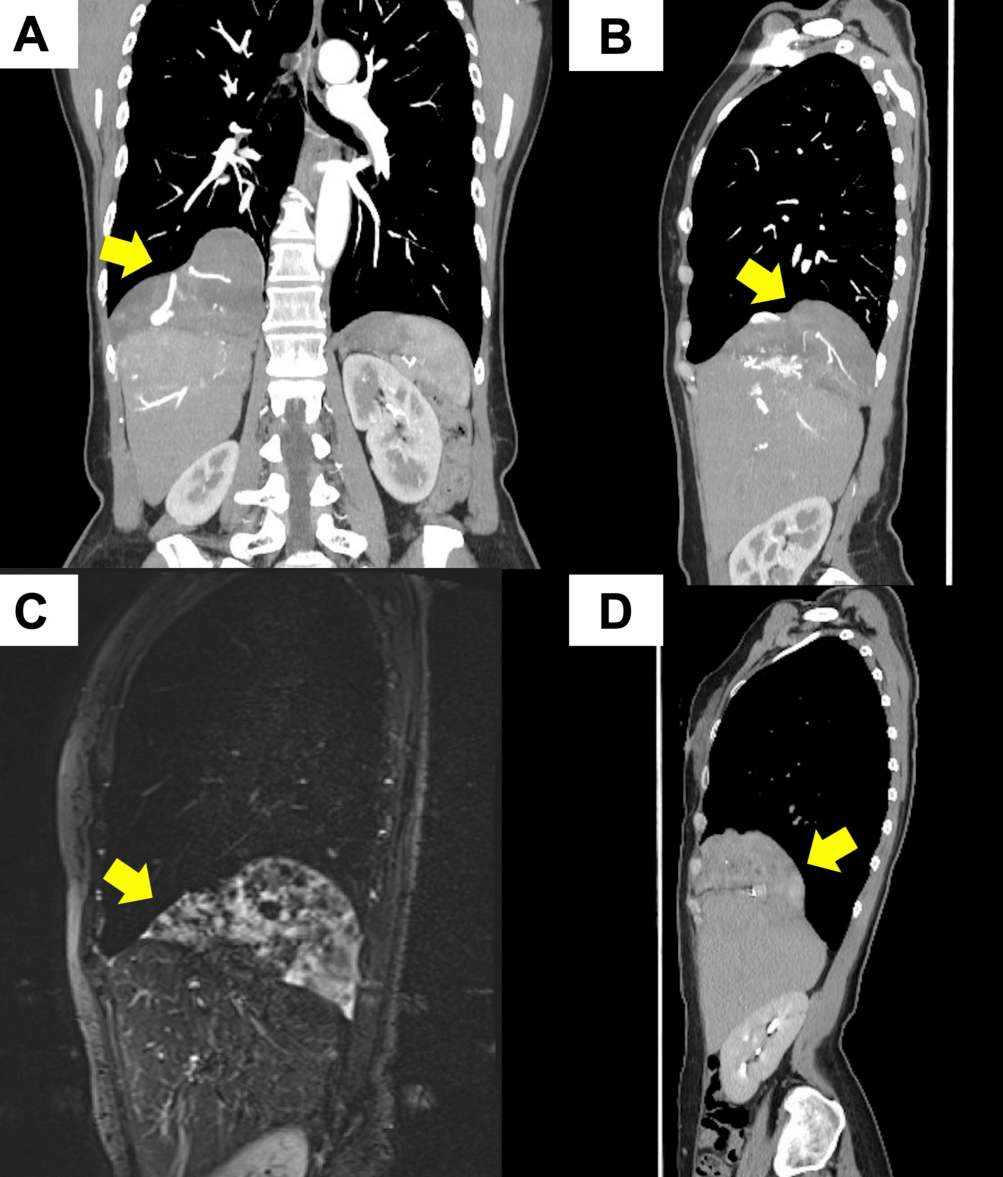
Preoperative imaging findings of the tumor (A, B) Mediastinal coronal and sagittal CT images in the supine position. (C) Mediastinal sagittal short-TI inversion recovery MRI image in the supine position. (D) The tumor is depressed ventrally in the prone position, and there is no invasion of the dorsal diaphragm or chest wall. The yellow arrow indicates the tumor.

A 60-year-old woman underwent computed tomography during a medical checkup, which revealed a 12.6 cm mass attached to the right hemidiaphragm, showing prominent vascularity with intermediate contrast enhancement (Figures 1A, 1B).

**Figure 2:**
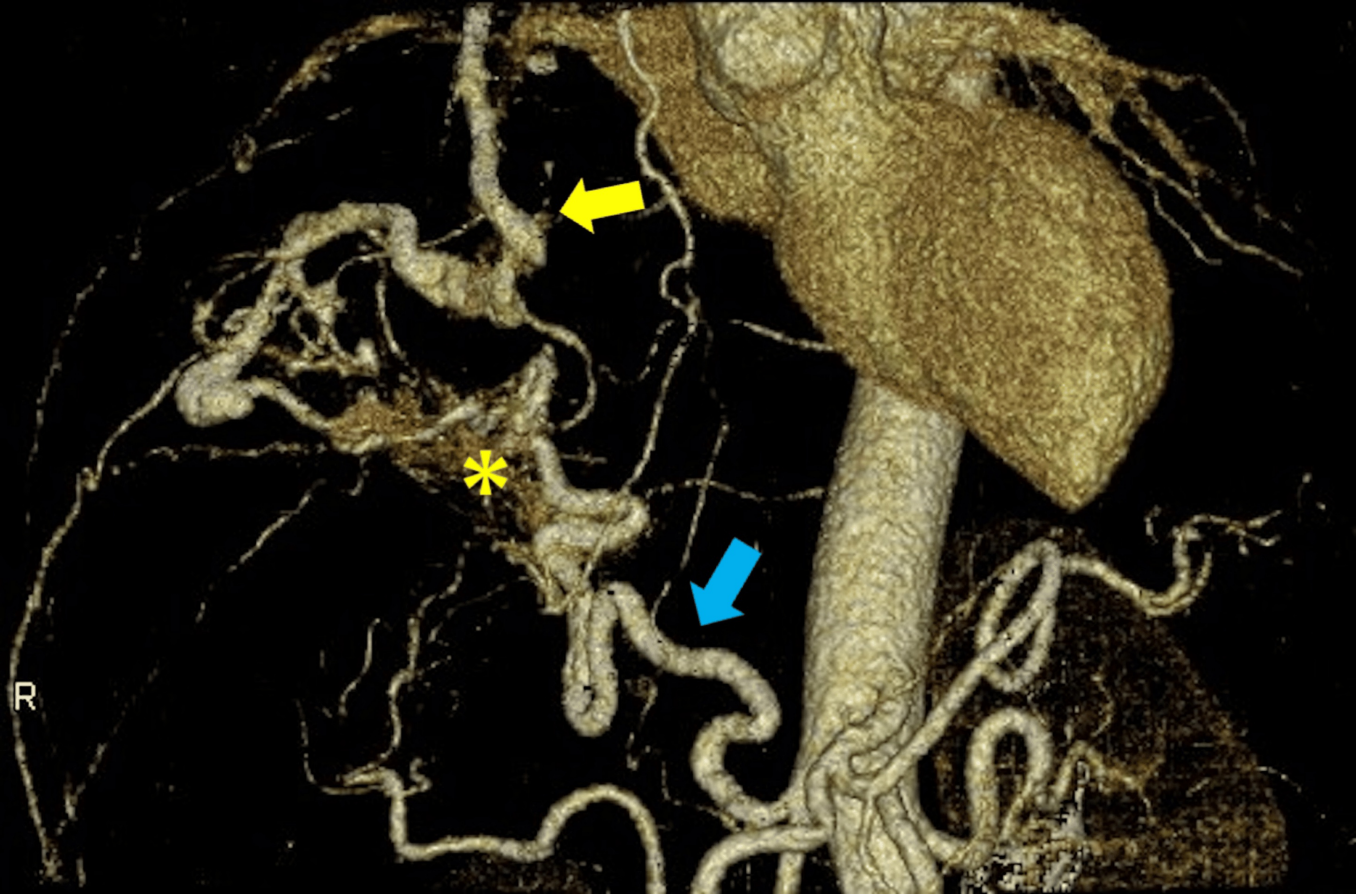
4D CT of the tumor Four-dimensional CT demonstrating the vascular supply to the tumor. The right inferior phrenic artery (blue arrow) arises directly from the abdominal aorta. The middle lobar artery (yellow arrow) is also one of the supplying vessels. The yellow asterisk (*) indicates vascular staining of the tumor.

Chest magnetic resonance imaging showed an extensive high signal intensity with patchy areas of low-signal intensity on short-TI inversion recovery (Figure 1C). A prone-position CT showed a ventrally depressed tumor, and there was no dorsal diaphragmatic or chest wall invasion (Figure 1D). Post-contrast imaging appearances were consistent with SFTPs, with angiogenic tumors such as hemangiopericytoma and hemangioendothelioma also considered as close differentials. On four-dimensional (4D) CT, the major vessel supplying the tumor was the right inferior phrenic artery, and a branch of the middle lobar artery also contributed as a supplying vessel. These vessels formed an arterio-arterial shunt (Figure 2; Appendix Video 1).

**Figure 3:**
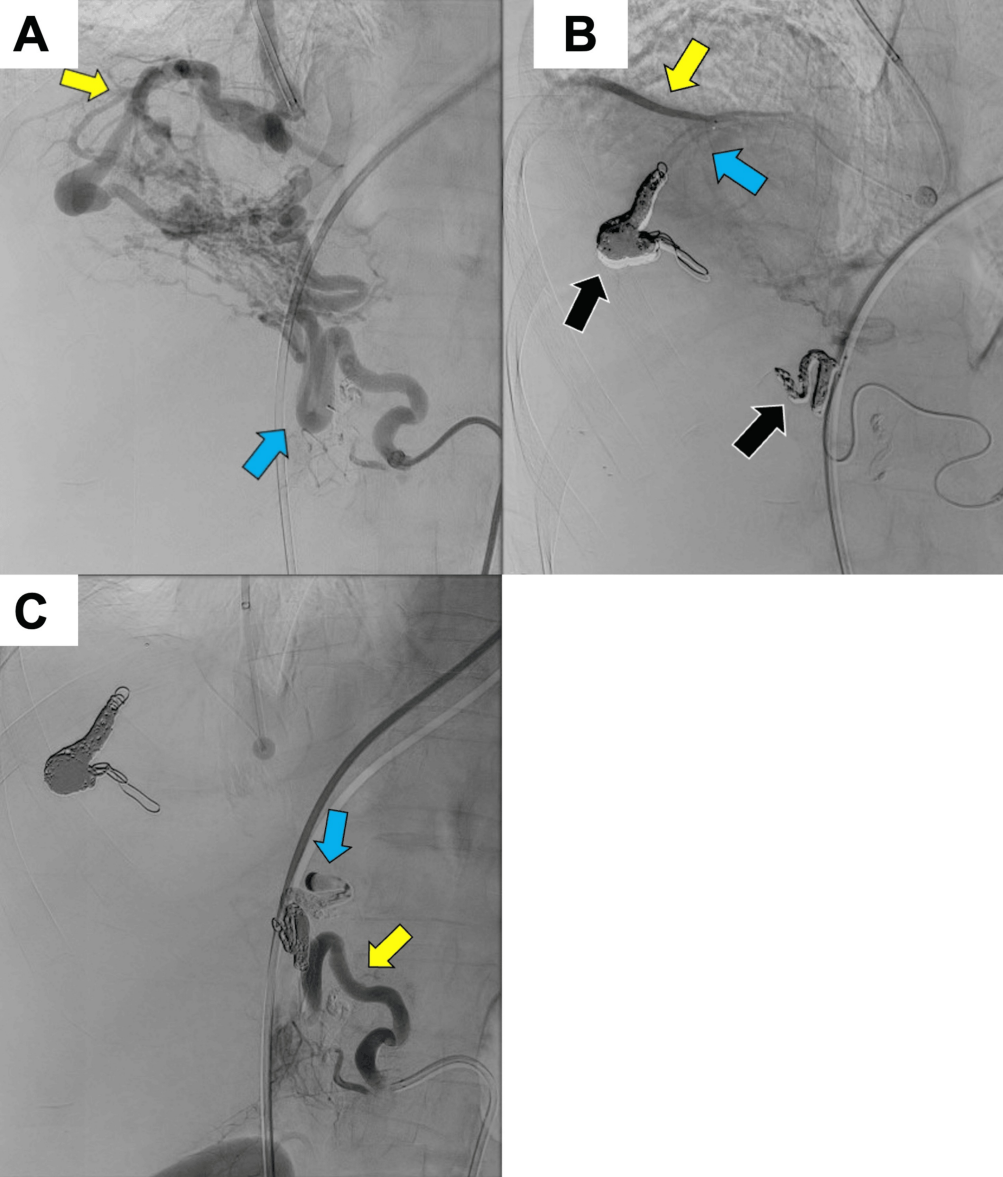
Angiography (A) The yellow and blue arrows show supplying vessels from the branches of the middle lobar and right inferior phrenic arteries, respectively. (B) The black arrows show the coils and embolization materials of the supplying vessels. The yellow arrow indicates that the cranial branch of the middle lobar artery is contrasted, and the blue arrow indicates that the caudal branch contrast is defective. (C) The yellow arrow indicates that the right inferior phrenic artery proximal to the coil is contrasted, and the blue arrow indicates that the central contrast is defective.

Additionally, twig-like branches from the right internal thoracic, intercostal, and hepatic arteries were appreciated on selective angiograms. Angiography followed by coil embolization was performed for all major arterial supplies, including the shunts (Figure 3).

**Figure 4:**
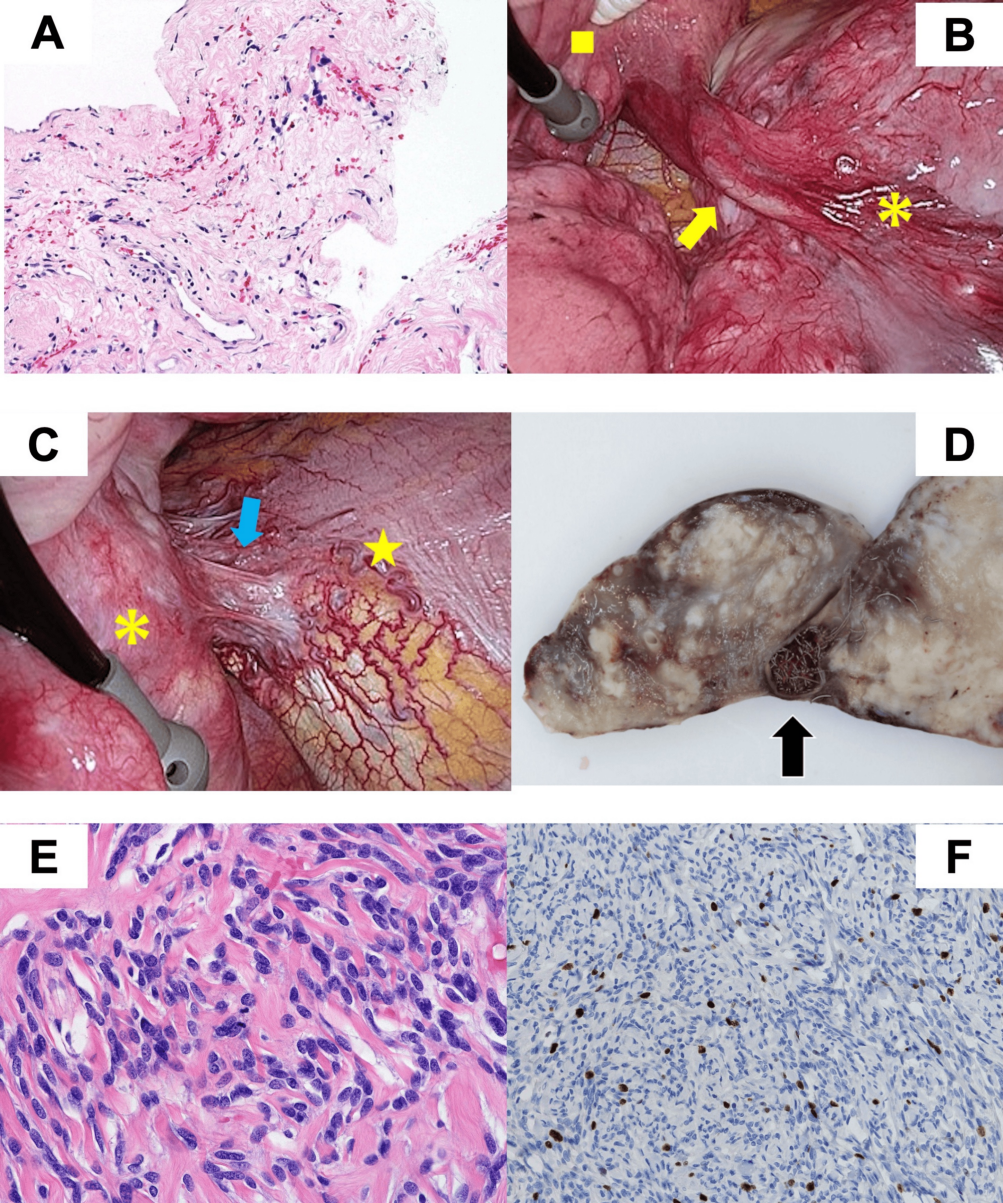
Intraoperative and pathological findings of the tumor (A) Ultrasound-guided percutaneous core needle biopsy specimen of the tumor showing features of SFTP (hematoxylin and eosin (HE) staining, high magnification). (B) The yellow arrow shows the supplying vessel from a branch of the middle lobar artery. The yellow asterisk indicates the tumor, and the yellow square indicates the right middle lobe. (C) The blue arrow shows adhesion of the tumor to the diaphragm, including the supplying vessel from the right inferior phrenic artery. The yellow star indicates the diaphragm. (D) Macroscopically, the tumor consists of a 16 cm solid mass surrounded by a thin membrane with an embolus inside (black arrow). (E and F) Microscopically, there were spindle-shaped atypical cells arranged in a storiform pattern, proliferating with alternating cellularity, with no necrosis, high cell density but poor nuclear atypia, and a fission image of 1–2 cells/10 with strong enlargement (E, HE staining, high magnification), and Ki-67 positive cell rate of approximately 5% (F, immunohistochemistry, high magnification). SFTP: Solitary fibrous tumor of the pleura

After confirming decreased blood flow, an ultrasound-guided percutaneous biopsy was performed, and a diagnosis of an SFTP was obtained (Figure 4A). Repeat pre-operative invasive angiography was performed to rule out other significant feeders, which were subsequently embolized.

Tumorectomy, combined with wedge resection of the right middle lobe, was performed using three-port video-assisted thoracoscopic surgery (VATS), as the cranial side of the tumor had invaded the right middle lobe (Figure 4B) (Appendix Video 2).

The caudal side showed abundant vascular growth (Figure 4C), which was dissected near the diaphragm to avoid tumor exposure. As there was no apparent tumor invasion into the diaphragm, combined diaphragmatic resection was not required. Minor oozing was controlled using soft coagulation. For specimen removal, the main port at the 5th intercostal space along the anterior axillary line was extended from 3 cm to 6 cm, and the 6th costal cartilage was transected to allow removal of the giant tumor (Appendix Video 3). Intraoperative frozen section diagnosis of the resected tumor revealed tumor-free surgical margins on both the right middle lobe and diaphragmatic side. The patient’s postoperative course was uneventful.

Macroscopically, the tumor comprised a 16 cm solid mass surrounded by a thin membrane with an embolus (Figure 4D). Histological examination revealed hemorrhage without necrosis, high cell density but poor nuclear atypia, and a fission image of 1-2 cells/10 with strong enlargement and a Ki-67 positive cell rate of approximately 5%, findings suggestive of a low-grade malignancy (Figures 4E, 4F). The lesion was diagnosed as an SFTP, which was consistent with the preoperative diagnosis.

## Discussion

This case highlights several crucial aspects of preoperative assessment and treatment of giant hypervascular tumors that extensively adhere to the diaphragm. First, the tumor size and widespread attachment to the diaphragm necessitated a precise preoperative diagnosis to define the correct surgical approach. The surgical plan could vary depending on the potential invasion of adjacent structures. Second, owing to the hypervascular nature of the tumor, preoperative arterial embolization was performed to reduce the blood supply of the tumor, enabling safe and minimally invasive resection. To address this issue, prone and supine CTs were performed to confirm the absence of contact. Furthermore, the incidental occurrence of pneumothorax during the preoperative biopsy provided additional confirmation that there was no IVC invasion, thereby allowing for a more conservative surgical plan. These steps emphasize the importance of comprehensive preoperative planning and embolization to optimize surgical outcomes in challenging cases.

SFTPs are rare neoplasms that account for <5% of primary pleural tumors. Patients are most commonly diagnosed between 50 and 70 years of age, with no clear gender predilection [10]. Epidemiologically, the estimated incidence is approximately 2.8 cases per 100,000 hospital patients [10]. SFTPs are derived from submesothelial mesenchymal layers, usually from visceral pleura, and rarely from parietal pleura [1,2]. Although approximately 86% of cases exhibit benign clinical behavior, approximately 13% of SFTPs are malignant [11]. Five-year overall survival after complete surgical resection ranges from 83% to 92% for benign SFTPs, with recurrence rates of approximately 8-11%; in contrast, malignant SFTPs have a higher recurrence rate (30-60%) and poorer outcomes [12]. Tapias et al. also reported an increased risk of recurrence when the tumor size was >10 cm [13,14]. Definitive diagnosis of malignant or benign tumors can be challenging with biopsy alone, particularly in large or hypervascular tumors where malignant degeneration is common. In the present case, intraoperative frozen section analysis of the resected tumor revealed no histopathological features suggestive of malignancy. Nevertheless, considering the potential for late recurrence, we plan long-term postoperative surveillance with periodic imaging.

We performed a literature review on patients with SFTPs who underwent preoperative arterial embolization. A literature search was conducted using the keywords “solitary fibrous tumor of the pleura” and “arterial embolization” in PubMed to identify associated or referenced articles. We identified 15 cases reported between 1998 and 2023. The key characteristics of these cases, including presenting symptoms, tumor size, main supplying arteries, and surgical approaches, are summarized in Table 1. 

**Table 1:** Summary of 15 patients who underwent surgical resection of SFTPs following arterial embolization SFTP: Solitary fibrous tumor of the pleura (a) Two of the included reports did not provide information on tumor size, and seven did not report tumor weight. (b) In some cases, the SFTP was supplied by multiple feeding arteries rather than a single vessel. (c) Two cases required both posterolateral thoracotomy and median sternotomy. Source: [2,7-9,15-21]

Variable	Category	Value
Sex	Men	6 (40%)
	Women	9 (60%)
Median age		59 (range: 37 - 78) years
Presenting symptoms (overlapping)	Breathlessness/Dyspnea	10 (66%)
	Chest pain	5 (33%)
	Cough	1 (6%)
	Fever	1 (6%)
	None	1 (6%)
Location of the SFTP	Right	9 (60%)
	Left	6 (40%)
Median tumor size (14 cases) (a)		21 (range: 15 - 33) cm
Median tumor weight (nine cases) (a)		3000 (range: 1425 - 4500) g
Feeding artery (overlapping) (b)	Inferior phrenic	4 (26%)
	Internal mammary	3 (20%)
	Abdominal aorta	2 (13%)
	Subclavian	2 (13%)
	Intercostal	2 (13%)
	Bronchial	1 (6%)
	Costocervical trunk	1 (6%)
	Thyrocervical	1 (6%)
	Hepatic	1 (6%)
Approach (overlapping) (c)	Thoracotomy	15 (100%)
	Median sternotomy	2 (13%)
Median bleeding (eight cases)		800 (range: 100 - 2365) ml

Fifteen cases of SFTPs were treated with arterial embolization in the preoperative period [2,7-9,15-21]. Breathlessness or dyspnea occurred in approximately 66% of the patients. The inferior phrenic and internal mammary arteries were the most frequently reported supplying arteries to the tumors, accounting for 27% and 20% of the patients, respectively. To the best of our knowledge, there have been no prior reports describing the formation of vascular shunts between systemic arteries supplying SFTPs. In the present case, all supplying arteries were systemic and not pulmonary arteries. All the reported cases were treated with thoracotomy, with two patients undergoing an additional median sternotomy. Median blood loss was 800 mL (range 100-2,365 mL). The median tumor size was 21 cm (range 15-33 cm), and giant tumors were common. No reported cases were managed with VATS, making this case report unique due to its minimally invasive approach. VATS was feasible in this case after preoperative embolization reduced vascularity, and no invasion into adjacent structures was found.

Although arterial embolization is an effective strategy for reducing intraoperative bleeding in hypervascular SFTPs, it carries risks that require careful management. Cases of fatal outcomes due to massive intraoperative bleeding during surgery for giant SFTPs have been reported, further emphasizing the need for preoperative embolization [22]. However, complications associated with arterial embolization must be carefully considered. Patel et al. described a 69-year-old woman with SFTP who developed a cerebral infarction during preoperative embolization of the right inferior phrenic artery [23]. The patient eventually developed respiratory failure, was intubated, and died the following day because her condition deteriorated due to a large infarct area throughout both the cerebral and cerebellar hemispheres.

Preoperative 4D CT is crucial for assessing vascular anatomy and planning an embolization strategy. Angiography identified other supplying vessels, including the right internal thoracic, intercostal, and hepatic arteries, which were embolized primarily to minimize residual vascularity rather than to prevent major intraoperative bleeding. Advanced imaging with time-resolved 4D CT and angiography enabled precise identification of vascular structures, allowing for a customized embolization approach. Notably, the involvement of a pulmonary artery shunt as a draining vessel in the SFTP, along with the successful complete resection by VATS, highlights the unique vascular characteristics of this case.

## Conclusions

This case is unique because of a distinct pulmonary artery shunt in a rare giant hypervascular SFTP. It highlights the importance of 4D CT in mapping the vascular anatomy, tailoring preoperative embolization, and planning minimally invasive surgery. Successful VATS resection with minimal bleeding highlights the importance of a thorough preoperative evaluation, as well as innovative imaging and image-guided interventions for managing such complex cases. This case provides valuable insights into the preoperative and intraoperative management of rare, complex SFTPs.
